# Nutrient gaps are prevalent among women experiencing infertility, a cross-sectional analysis of the National Health and Nutrition Examination Survey (NHANES), 2013–2020

**DOI:** 10.1371/journal.pone.0341444

**Published:** 2026-01-27

**Authors:** Rachel A. Murphy, Carroll A. Reider, Ryan W. Grant, Susan Hazels Mitmesser

**Affiliations:** 1 School of Population and Public Health, University of British Columbia, Vancouver, British Columbia, Canada; 2 Population Health Sciences, BC Cancer, Vancouver, British Columbia, Canada; 3 Science and Technology, Pharmavite LLC, West Hills, California, United States of America; Universiti Sains Malaysia, MALAYSIA

## Abstract

**Background:**

Adequate nutrient intake is important for supporting reproductive health. Few studies have examined the role of nutrients for fertility among women, resulting in a critical evidence gap. The aim of this study was to explore the usual intake and prevalence of nutrient inadequacies from foods only and foods plus dietary supplements among women of child bearing age with and without infertility.

**Methods:**

This cross-sectional study used data from the National Health and Nutrition Examination Survey (NHANES), a national survey in the United States. Participants included women aged 18–44 years from 2013–2020 with or without infertility (difficulty trying to conceive for at least one-year). The mean usual intakes and prevalence of inadequacy (% < EAR/AI) of key nutrients- vitamins A, B1, B2, niacin, B6, B12, C, D, E, K, lycopene, lutein + zeaxanthin, folate, choline, zinc, selenium, iron, calcium, magnesium, EPA and DHA were determined from 24-hr dietary recalls.

**Results:**

Women aged 18–44 years who reported infertility had significantly lower intakes of vitamin A, E and K and lutein and zeaxanthin from foods and foods + supplements compared to women without infertility. Lower intakes of selenium (foods only, 96.6 ug/d vs 100 ug/d), vitamin C (foods only, 66.9 mg/d vs. 74.2 mg/d) and calcium (foods + dietary supplements, 941 mg/d vs. 974 mg/d) were also observed in women reporting infertility. Fewer women with infertility met nutrient requirements. For example, 21.5% of women with infertility were below the EAR for vitamin B6 versus 14.6% of women without infertility and over 50% of those with infertility were below the EAR/AI for vitamins A, C, E, and magnesium and potassium. Differences in nutrient intakes by fertility status were particularly pronounced for women aged 35–44 years.

**Conclusions:**

These findings suggest lower intake of key nutrients among women with infertility, especially those aged 35–44. Future studies are needed to understand the implications of nutrient gaps for conception. Across all age groups, and fertility status, gaps in nutrients that are important for overall health were evident, underscoring the need for public health strategies to address broad dietary improvements.

## Background

Global estimates from the World Health Organization highlight the rising incidence of infertility, the inability to conceive after one year of unprotected sex [1]. In the United States, infertility affects millions of men and women [2]; an estimated 10.43 million women aged 15–49 years experienced infertility in 2015–2019 [2]. Infertility is particularly prevalent among women aged 40 and older, those with lower levels of education and non-Hispanic black women [3]. The magnitude of infertility, and by extension, substantive population in need of fertility care, is a pressing public health concern.

In 2014, the United States Centers for Disease Control and Prevention released the National Public Health Action Plan for the detection, prevention, and management of infertility [4]. Among other actions, the plan highlighted the need for programs aimed at behavioral risk factors for infertility, including dietary intake. As dietary intake is modifiable with minimal side effects versus pharmacological interventions, programs that target dietary/nutrient intake are a promising opportunity to support reproductive health. However, to inform such programs, specific dietary guidance is needed, as is an understanding of the nutrients needed to support fertility among women of childbearing age. Studies have typically focused on select nutrients such as those with antioxidant properties (e.g., vitamins A, C, E, and carotenoids) [5] and are heterogenous in design and population [6], such as couples receiving assisted reproductive treatment [5]. This limits the generalizability of findings to public health dietary guidance. Little is known regarding possible benefits of a broader range of nutrients for fertility. Given that foods and indeed, many supplements are complex combinations of nutrients, it is critical to understand the potential role of commonly consumed nutrients in relation to fertility.

The National Health and Nutrition Examination Survey (NHANES) which is nationally representative of the United States population, routinely assesses dietary intake. Over the course of seven years (2013–2020), NHANES captured information on fertility through self-assessed difficulty trying to conceive. This provides the opportunity to conduct an exploratory study to provide new insight into the nutritional needs of women with respect to fertility. This study aimed to explore the usual intake of nutrients – vitamins A, B1, B2, niacin, B6, B12, C, D, E, K, lycopene, lutein and zeaxanthin, folate, choline, zinc, selenium, iron, calcium, magnesium, EPA and DHA among women who did and did not report infertility. Secondary analyses additionally explored associations between dietary intake and difficulty trying to conceive among women aged 18–34 years and 35–44 years.

## Methods

The NHANES assesses the health and nutritional status of children and adults in the United States. NHANES generally occurs in two-year cycles, with the exception of the 2019–2020 cycle that was suspended due to the coronavirus disease. Data from NHANES 2017–2018, 2019–2020 and data collected from 2019 to March 2020 were combined to form a nationally representative sample from 2017 to 2020. The data was accessed November 15, 2023. The NHANES protocol was approved by the National Center for Health Statistics Research Ethics Review Board. Participants provided written informed consent. As this analysis uses publicly available data, ethics approval from the authors Institution was not required. The multistage probability sampling design of NHANES provides nationally representative estimates for the civilian population in the United States. Data collection in the NHANES includes a household interview during which time, survey personnel collect information on supplement use in the prior 30 days. Trained interviewers also collect detailed dietary information on all foods, beverages and dietary supplements consumed in the prior 24 hours (day 1). Between 3 to 10 days later, a second 24-hour dietary recall (day 2) is administered by phone on a different day of the week.

### Study population

The sample population for this study included women aged 19–44 years who participated in the What We Eat in America component of the NHANES. Women who completed the reproductive health questionnaire component of NHANES were also included. Prior to 2018 and after 2020, fertility was not consistently captured. Therefore, we used data from cycles 2013–2014, 2015–2016, and 2017–2020, henceforth referred to as 2013–2020. Women who reported being pregnant or breastfeeding were excluded due to differences in nutrient requirements as were women who had a dietary recall that was not reliable (DR1DRSTZ=1). Women who did not complete the reproductive questionnaire that queried fertility, and who did not complete the dietary supplement questionnaire were also excluded.

### Nutrient consumption

The estimated consumption of nutrients from foods and beverages was based on the 24-hour dietary recalls and corresponding nutrient composition data from the Food and Nutrient Database for Dietary Studies. The contribution of dietary supplements to nutrient intake was estimated using dietary supplement intake during the prior 30 days. The bioavailability of folate in food is estimated to be lower than bioavailability of folic acid in dietary supplements and fortified foods. The dietary folate equivalent conversion was used to estimate nutrient adequacy. Carotenoids with retinol activity were used to estimate vitamin A intake and AI. Data on vitamins A and E in dietary supplements between 2007 to 2018 were estimated using previous databases of products as more contemporary information is not available in the NHANES.

### Fertility

Fertility was self-reported from the question “Tried for a year to become pregnant?” Women who answered ‘yes’ were classified as experiencing infertility, while ‘no’ was classified as not experiencing infertility.

### Statistical analysis

The usual intake of nutrients from foods and beverages was estimated using the National Cancer Institute method and corresponding National Cancer Institute developed SAS macros. Nutrients were modeled using DISTRIB; a single component model that is appropriate for nutrients consumed by most participants each day. EPA and DHA were modeled with MIXTRAN; a two-part correlated model since EPA and DHA are episodically consumed. Statistical models included age, weekday (Mon to Thu)/weekday (Fri to Sun), sequential effect of recall (first or second), and a variable indicating use of dietary supplements (yes/no). The distribution of total usual nutrient intake from all sources was determined using the shrink then add approach described previously [7]. This approach models supplement use as an indicator variable and subsequently adds the usual intake from dietary supplements to the distribution of nutrient intakes from foods and beverages. The cut-point approach [8] was used to determine the prevalence of the population that met age-specific Estimated Average Requirements (EAR) or the Adequate Intake (AI). In the absence of an EAR or AI for EPA and DHA, the prevalence of the population with intake below 250 mg/d, the recommendation from the Dietary Guidelines for Americans was used [9]. Differences in the usual intake of nutrients between women with and without infertility were determined by Student’s t-tests. Chi-square tests were used to determine differences between the proportion of women above/below the EAR or AI. Statistical significance was set at p < 0.05. Sampling weights were averaged across all two-year cycles, with adjustment for the longer period and larger population in the 2017-March 2020 files as recommended [10]. Sampling units and strata provided by the NHANES were included in all analyses. Estimates with a relative SE greater than 30% are not presented in accordance with the National Center for Health Statistics analytical guidelines. Statistical analysis was performed using SAS v.9.4 (SAS Institute, Cary, NC, USA).

## Results

A total of 3,900 women were included in this study (Table 1), including 412 who reported infertility. Compared to women who did not report infertility, women with infertility were predominately white (58%), older, had more than a high school education (67%), and an obese BMI (57%). Overall, nearly 50% of women reported dietary supplement use. The usual intake of nutrients from foods and dietary supplements was generally higher than foods only (Tables 3–5), with the exception of vitamin K which is infrequently included in dietary supplements. The intake of EPA and DHA, was universally low and as a result, the prevalence of inadequate intake was above 93% for all women.

**Table 1:** Study population characteristics of women aged 18-44y, NHANES 2013-2020.

	Infertility		
	Total	Yes	No
n	3,900	412	3,488
Age, mean (SE), years	31.0 (0.18)	34.4 (0.44)	30.6 (0.18)*
Race/ethnicity, %			
Mexican American	644 (11.9)	66 (11.7)	578 (11.9)
Other Hispanic	423 (8.21)	40 (7.88)	383 (8.25)
Non-Hispanic white	1244 (56.2)	143 (58.4)	1101 (55.9)
Non-Hispanic black	946 (13.5)	98 (13.2)	848 (13.5)
Other race (including multiracial)	643 (10.2)	65 (8.85)	578 (10.4)
Household income, %			
< 1.35 of PIR	1461 (30.8)	141 (30.5)	1320 (30.8)
1.35–1.85 of PIR	526 (13.0)	46 (11.1)	480 (13.3)
> 1.85 of PIR	1629 (56.2)	191 (58.4)	1438 (55.9)
Education, %			
≤ high school	1203 (30.4)	149 (33.3)	1054 (30.0)
> high school	2246 (69.6)	252 (66.7)	1994 (70.0)
BMI, %			
Underweight	114 (2.67)	6 (1.82)	108 (2.78)*
Normal	1240 (33.5)	100 (23.5)	1140 (34.7)
Overweight	913 (23.6)	73 (18.0)	840 (24.3)
Obese	1633 (40.3)	233 (56.8)	1400 (38.2)
Dietary supplement use, %	1764 (49.6)	190 (47.4)	1574 (49.8)

PIR; poverty to income ratio. * Indicates mean differences p < 0.05 between adjacent columns.

Among women aged 18-44y, there were several nutrients that had a significantly lower intake and higher nutrient inadequacy in those with reported infertility than those without reported infertility (p < 0.05, Table 2). Women who reported infertility had lower intakes of vitamins A, E and K and lutein and zeaxanthin from food and foods + supplements. For example, the mean (SE) of vitamin A from foods only was 506 (27.0) vs. 581 (13.5) RAE/d, while the mean (SE) of vitamin E from foods only was 8.16 (0.33) vs. 8.75 (0.18). Lower intake of selenium (foods only), vitamin C (foods only) and calcium (foods + dietary supplements) were also observed in women who reported infertility. The risk of nutrient inadequacy (proportion below the EAR) among women aged 18–44 y with infertility was higher compared to those without infertility for vitamin A (56.2% vs 44.0% for foods only, Table 3). Similarly, the risk of vitamin B6, vitamin C, vitamin E, vitamin K, magnesium and potassium inadequacy was higher among women with infertility from foods only and for foods + dietary supplements. The proportion at risk of vitamin E inadequacy was notably high among women with infertility (foods only: 87.3% vs. 83.5%, Table 3).

**Table 2:** Usual intake of nutrients in women aged 18-44y with and without infertility, NHANES 2013-2020.

	Foods only		Foods + supplements	
	Infertility		Infertility	
Nutrient, mean (SE)	Yes, N = 412	No, N = 3,488	Yes, N = 412	No, N = 3,488
Vitamin A, RAE/d	506 (27.0)	581 (13.5)*	584 (40.8)	692 (22.6)*
Thiamin, mg/d	1.38 (0.04)	1.40 (0.02)	2.63 (0.43)	2.88 (0.18)
Riboflavin, mg/d	1.80 (0.06)	1.82 (0.03)	2.94 (0.41)	3.05 (0.12)
Niacin, mg/d	22.3 (0.69)	22.7 (0.24)	28.3 (2.06)	28.1 (0.60)
Vitamin B6, mg/d	1.76 (0.07)	1.88 (0.03)	3.30 (0.36)	4.04 (0.34)
Vitamin B12, ug/d	3.90 (0.17)	4.02 (0.08)	39.8 (14.2)	49.2 (7.65)
Vitamin C, mg/d	66.9 (3.85)	74.2 (2.13)*	140 (33.4)	131 (7.33)
Vitamin D, mg/d	3.68 (0.19)	3.77 (0.11)	19.5 (2.39)	16.3 (0.82)
Vitamin E, mg/d	8.16 (0.33)	8.75 (0.18)*	14.8 (1.20)	19.2 (1.27)*
Vitamin K, μg/d	109 (6.47)	123 (4.82)*	112 (6.58)	129 (4.96)*
Zinc, mg/d	9.34 (0.34)	9.54 (0.12)	12.3 (0.60)	11.9 (0.20)
Iron, mg/d	11.8 (0.44)	12.3 (0.13)	15.7 (0.71)	16.6 (0.29)
Choline, mg/d	277 (9.63)	286 (3.66)	280 (11.0)	289 (3.62)
Folate, μg DFE/d	438 (19.1)	455 (6.50)	681 (46.8)	640 (12.1)
Calcium, mg/d	866 (24.4)	879 (9.72)	941 (26.0)	974 (12.9)*
Copper, mg/d	1.09 (0.03)	1.09 (0.02)	1.26 (0.05)	1.28 (0.02)
Selenium, μg/d	96.6 (2.90)	100 (1.01)*	102 (3.43)	107 (1.32)
Phosphorus, mg/d	1214 (30.2)	1220 (11.8)	1215 (30.3)	1223 (11.9)
Potassium, mg/d	2195 (55.1)	2277 (30.8)	2199 (55.0)	2283 (30.6)
Magnesium, mg/d	262 (6.99)	270 (3.66)	276 (8.36)	286 (4.30)
EPA + DHA, mg/d	66.9 (5.07)	65.4 (3.56)	90.6 (9.20)	89.8 (4.66)
Lutein + zeaxanthin, μg/d	1424 (104)	1604 (84.8)*	1439 (105)	1654 (90.2)*

* Indicates mean differences p < 0.05 between adjacent columns.

**Table 3:** Percentage of women with and without infertility with usual intake from foods only and food and supplements <EAR or <AI, aged 18-44y, NHANES 2013-2020.

Nutrient	Foods only		Foods + supplements	
	Infertility		Infertility	
	Yes, N = 412	No, N = 3,488	Yes, N = 412	No, N = 3,488
% < EAR				
Vitamin A	56.2 (4.53)	44.0 (1.93)*	55.3 (4.70)	43.4 (1.87)*
Thiamin	10.7 (2.15)	9.37 (1.49)	9.27 (1.90)	7.90 (1.25)
Riboflavin	4.51 (1.06)	4.16 (0.77)	4.04 (0.95)	3.63 (0.67)
Niacin	2.21 (0.78)	1.83 (0.49)	1.87 (0.66)	1.51 (0.41)
Vitamin B6	21.5 (4.02)	14.6 (1.73)*	16.8 (3.20)	11.4 (1.36)*
Vitamin B12	10.9 (2.55)	9.56 (1.33)	8.44 (2.11)	7.04 (0.98)
Vitamin C	52.2 (3.91)	44.9 (2.26)*	42.2 (3.59)	35.0 (1.91)*
Vitamin D	98.6 (0.46)	98.5 (0.40)	66.9 (2.64)	68.0 (0.97)
Vitamin E	87.3 (2.39)	83.5 (1.93)*	71.1 (2.89)	68.0 (1.85)*
Zinc	54.6 (5.20)	51.4 (1.72)	44.5 (4.65)	42.2 (1.48)
Iron	15.1 (3.40)	11.8 (1.39)	12.5 (2.28)	10.6 (1.05)
Folate	22.8 (3.51)	19.6 (2.18)	18.7 (3.02)	15.6 (1.76)
Calcium	44.0 (3.95)	41.7 (1.58)	38.0 (3.67)	35.0 (1.50)
Copper	12.7 (1.84)	12.2 (1.04)	11.8 (1.73)	11.2 (0.95)
Selenium	1.10 (0.60)	0.78 (0.29)	1.05 (0.57)	0.71 (0.26)
Phosphorus	0.95 (0.40)	0.87 (0.28)	0.94 (0.40)	0.86 (0.28)
Magnesium	54.0 (3.61)	48.7 (1.74)*	51.2 (3.48)	44.9 (1.70)*
% < AI				
Vitamin K	45.8 (4.95)	36.5 (3.24)*	44.1 (4.83)	34.5 (3.06)*
Choline	94.6 (1.37)	93.2 (0.89)	94.0 (1.65)	92.8 (0.90)
Potassium	76.1 (2.92)	71.9 (1.89)*	75.9 (2.91)	71.6 (1.88)*
EPA + DHA	96.3 (0.71)	96.4 (0.57)	92.9 (1.23)	93.5 (0.59)
Lutein + zeaxanthin	99.2 (0.29)	98.7 (0.39)	99.1 (0.30)	98.3 (0.48)

AI; Adequate Intake, EAR; Estimated Average Requirement, DHA; docosahexaenoic acid, EPA; eicosapentaenoic acid, RAE; retinol activity equivalents. AI for EPA + DHA 250 mg, AI for lutein+ zeaxanthin 6,000 μg/d. *Indicates mean differences p < 0.05 between adjacent columns.

When examined by age subgroups, differences in nutrient intake between women with and without infertility among those aged 18–34 were only apparent for vitamin A: 553 (38.5) vs. 688 (27.0) for foods and dietary supplements (Table 4). Conversely, nutrient intakes among women aged 35–44 years with infertility were lower for vitamin A, potassium and magnesium from foods and foods plus dietary supplements. The percentage of women at risk of shortfalls in vitamin A, B6, C, D, E, and K, magnesium and potassium were all greater among women with infertility, aged 35–44 years (Table 5).

**Table 4:** Usual intake of nutrients in women aged 18-44y with and without infertility by age, NHANES 2013-2020.

Nutrient	Foods only				Foods + supplements			
	Infertility				Infertility			
	18-34y		35-44y		18-34y		35-44y	
	Yes N = 198	No N = 2280	Yes N = 214	No N = 1208	Yes N = 198	No N = 2280	Yes N = 214	No N = 1208
Vitamin A, RAE/d	507 (34.3)	578 (16.2)	493 (44.1)	585 (24.0)*	553 (38.5)	688 (27.0)*	601 (69.6)	699 (31.4)*
Thiamin, mg/d	1.42 (0.07)	1.41 (0.02)	1.32 (0.06)	1.39 (0.03)	2.39 (0.51)	2.68 (0.27)	2.83 (0.66)	3.28 (0.20)
Riboflavin, mg/d	1.71 (0.07)	1.79 (0.03)	1.85 (0.09)	1.87 (0.04)	2.62 (0.46)	2.78 (0.15)	3.20 (0.64)	3.58 (0.26)
Niacin, mg/d	22.5 (0.78)	22.7 (0.31)	21.9 (1.15)	22.6 (0.42)	29.1 (3.44)	27.4 (0.84)	27.2 (1.72)	29.3 (0.73)
Vitamin B6, mg/d	1.78 (0.08)	1.87 (0.04)	1.72 (0.11)	1.89 (0.05)	3.02 (0.40)	3.75 (0.48)	3.55 (0.55)	4.60 (0.45)
Vitamin B12, ug/d	3.80 (0.20)	4.00 (0.08)	3.90 (0.31)	4.04 (013)	12.3 (5.49)	41.4 (10.4)	64.9 (27.5)	64.3 (9.52)
Vitamin C, mg/d	63.2 (5.21)	74.7 (2.48)	67.3 (5.59)	73.1 (2.70)	159 (69.0)	115 (6.53)	120 (12.1)	164 (19.4)
Vitamin D, mg/d	3.81 (0.28)	3.72 (0.12)	3.48 (0.31)	3.86 (0.20)	12.4 (2.18)	10.4 (0.88)	17.5 (7.23)	15.9 (1.29)
Vitamin E, mg/d	8.10 (0.39)	8.64 (0.21)	8.16 (0.60)	8.93 (0.31)	13.2 (1.42)	16.8 (1.33)	16.3 (1.85)	23.8 (2.32)
Vitamin K, μg/d	107 (10.1)	119 (5.97)	107 (11.2)	129 (5.84)	108 (10.2)	123 (5.98)	112 (11.3)	139 (6.54)
Zinc, mg/d	9.46 (0.46)	9.51 (0.14)	9.09 (0.51)	9.60 (0.22)	12.2 (0.81)	11.7 (0.25)	12.3 (0.75)	12.5 (0.37)
Iron, mg/d	11.9 (0.57)	12.3 (0.21)	11.5 (0.69)	12.3 (0.24)	14.1 (0.93)	16.4 (0.37)	16.4 (1.20)	17.0 (0.70)
Choline, mg/d	279 (11.4)	284 (4.89)	270 (15.4)	290 (6.05)	279 (11.4)	285 (4.95)	275 (17.2)	298 (5.84)
Folate, μg DFE/d	455 (29.8)	457 (9.60)	414 (23.7)	450 (9.87)	663 (74.6)	628 (15.8)	687 (51.5)	664 (21.0)
Calcium, mg/d	858 (33.1)	882 (13.0)	858 (37.3)	872 (19.0)	899 (34.2)	954 (14.4)	965 (41.0)	1014 (30.1)
Copper, mg/d	1.06 (0.04)	1.08 (0.02)	1.09 (0.05)	1.12 (0.02)	1.18 (0.06)	1.25 (0.03)	1.32 (0.07)	1.33 (0.03)
Selenium, μg/d	96.5 (3.57)	100 (1.24)	95.6 (4.82)	100 (1.82)	99.0 (3.79)	105 (1.43)	103 (5.76)	110 (2.58)
Phosphorus, mg/d	1194 (37.9)	1222 (15.5)	1213 (47.9)	1215 (20.3)	1195 (38.0)	1224 (15.4)	1215 (48.0)	1220 (20.6)
Potassium, mg/d	2175 (58.9)	2263 (39.6)	2167 (95.8)	2302 (40.5)*	2179 (58.6)	2266 (39.6)	2171 (96.2)	2311 (40.0)*
Magnesium, mg/d	259 (9.18)	265 (4.82)	260 (11.7)	279 (4.64)*	278 (11.7)	277 (5.41)	270 (12.3)	305 (6.40)*
EPA + DHA, mg/d	66.0 (6.45)	64.8 (4.48)	54.5 (7.20)	62.9 (5.17)	78.8 (9.99)	83.9 (4.95)	88.3 (14.1)	97.5 (9.03)
Lutein + zeaxanthin, μg/d	1508 (153)	1554 (105)	1289 (180)	1684 (103)	1512 (153)	1591 (106)	1314 (171)	1760 (118)

* Indicates mean differences p < 0.05 between adjacent columns.

**Table 5:** Percentage of women with and without infertility with usual intake from foods only and food and supplements <EAR or <AI, by age, NHANES 2013-2020.

Nutrient	Foods only				Foods + supplements			
	Infertility				Infertility			
	18-34y		35-44y		18-34y		35-44y	
	Yes N = 198	No N = 2280	Yes N = 214	No N = 1208	Yes N = 198	No N = 2280	Yes N = 214	No N = 1208
% < EAR								
Vitamin A	55.6 (5.63)	44.5 (2.53)	58.0 (7.74)	42.1 (2.96)*	54.2 (5.66)	43.9 (2.37)*	57.0 (7.41)	42.5 (3.13)*
Thiamin	7.78 (2.28)	8.33 (1.81)	14.9 (4.19)	11.5 (2.17)	7.08 (2.11)	7.22 (1.57)	12.5 (3.60)	9.44 (1.73)
Riboflavin	5.49 (1.77)	4.01 (1.00)	4.50 (1.63)	4.13 (1.03)	5.09 (1.67)	3.58 (0.90)	3.81 (1.39)	3.49 (0.84)
Niacin	1.64 (0.68)	1.59 (0.60)	2.89 (1.64)	2.13 (0.90)	1.45 (0.58)	1.32 (0.52)	2.31 (1.40)	1.66 (0.70)
Vitamin B6	15.7 (2.91)	11.6 (1.84)	28.1 (7.56)	19.9 (3.33)*	12.2 (2.16)	9.35 (1.53)	21.6 (5.96)	15.0 (2.37)*
Vitamin B12	10.4 (3.04)	8.67 (1.69)	13.1 (4.91)	11.3 (1.83)	8.37 (2.58)	6.40 (1.28)	9.96 (3.87)	8.14 (1.34)
Vitamin C	56.5 (5.05)	45.8 (2.66)	50.7 (6.56)	43.9 (3.13)*	47.9 (4.99)	37.2 (2.42)	38.3 (5.15)	31.2 (2.36)*
Vitamin D	98.3 (0.80)	98.4 (0.51)	99.2 (0.60)	98.6 (0.68)	75.7 (4.18)	76.1 (1.30)	73.9 (3.08)	67.8 (2.03)*
Vitamin E	89.0 (3.00)	85.1 (1.97)	86.0 (4.10)	80.8 (3.01)	73.6 (4.20)	70.5 (1.96)	69.4 (4.10)	63.6 (2.91)
Zinc	52.3 (7.06)	51.8 (2.09)	58.3 (7.61)	50.9 (3.08)	44.3 (6.34)	43.7 (1.87)	45.2 (6.36)	40.0 (2.50)
Iron	13.5 (4.28)	11.4 (1.93)	17.7 (5.60)	12.8 (2.21)	12.3 (4.01)	9.83 (1.67)	15.0 (4.82)	10.9 (1.82)
Folate	19.6 (4.09)	19.4 (2.98)	28.1 (5.92)	20.5 (2.26)	16.4 (3.67)	15.8 (2.45)	22.4 (4.93)	15.7 (1.68)
Calcium	44.3 (5.15)	41.4 (2.12)	45.4 (6.11)	42.6 (2.79)	39.7 (4.94)	35.9 (2.04)	37.7 (5.30)	33.8 (2.35)
Copper	14.8 (2.60)	13.6 (1.45)	11.0 (2.86)	9.53 (1.69)	14.1 (2.52)	12.8 (1.36)	9.84 (2.56)	8.39 (1.38)
Selenium	0.82 (0.50)	0.56 (0.33)	1.56 (1.35)	1.07 (0.54)	0.81 (0.49)	0.53 (0.31)	1.36 (1.28)	0.92 (0.48)
Phosphorus	0.93 (0.50)	0.76(0.35)	0.78 (0.69)	0.94 (0.40)	0.93 (0.49)	0.75 (0.35)	0.75 (0.70)	0.92 (0.40)
Magnesium	53.4 (4.58)	50.1 (2.23)	56.0 (6.25)	46.2 (2.55)*	51.2 (5.47)	47.2 (2.22)	52.2 (6.07)	41.0 (2.32)*
% < AI								
Vitamin K	46.7 (6.79)	38.7 (4.10)	48.1 (8.78)	33.7 (3.62)*	45.9 (6.71)	37.2 (3.91)	45.0 (8.45)	30.6 (3.44)*
Choline	94.5 (1.96)	93.7 (1.11)	95.3 (1.84)	92.4 (1.85)	94.5 (1.97)	93.4 (1.12)	94.3 (2.30)	91.8 (1.84)
Potassium	77.8 (3.37)	72.6 (2.35)	77.5 (4.82)	70.4 (2.44)*	77.6 (3.36)	72.4 (2.36)	77.3 (4.87)	70.0 (2.43)*
EPA + DHA	95.8 (0.96)	95.8 (0.74)	99.5 (0.39)	99.1 (0.48)	94.6 (1.11)	93.4 (0.75)	93.8 (2.00)	95.1 (0.79)
Lutein + zeaxanthin	99.1 (0.64)	98.9 (0.44)	99.4 (0.34)	98.4 (0.60)	99.1 (0.64)	98.6 (0.46)	99.4 (0.35)	97.6 (0.88)

AI; Adequate Intake, EAR; Estimated Average Requirement, DHA; docosahexaenoic acid, EPA; eicosapentaenoic acid, RAE; retinol activity equivalents. AI for EPA + DHA 250 mg, AI for lutein+ zeaxanthin 6,000 μg. *Indicates mean differences p < 0.05 between adjacent columns.

## Discussion

The importance of a healthy diet that meets the nutritional requirements of women throughout life is well documented [11]. The DRIs already recognize unique nutrient requirements for pregnancy and lactation but the potentially unique nutritional needs of women during the pre-conception period are not well-defined. Our findings suggest lower intakes of multiple nutrients among women who reported infertility: vitamins A, E and K and lutein and zeaxanthin, and subsequently, higher risk of multiple inadequacies for the aforementioned nutrients in addition to vitamins B6 and C, magnesium and potassium. Differences in nutritional status were particularly evident for women who are later in childbearing age (35–44 years), when fertility begins to decline.

Preclinical and clinical studies provide biological plausibility to support these findings through direct (i.e., antioxidant effects) or downstream effects. For example, findings from pre-clinical models may be relevant to the observation of lower vitamin A among women who reported infertility. Animal models have demonstrated the importance of vitamin A for reproduction in males and females [12,13] whereby during deficiency spermatogenesis is arrested (among other effects in the testes), and in female rats, ovulation occurs irregularly with impairments to blastogens [13]. When vitamin A is provided in limited quantity, fertilization and implantation occur, but embryonic death is common [14]. Studies on lutein and zeaxanthin and fertility are limited, although insights can be drawn from epidemiologic studies of carotenoids. In a cohort of 1,228 women, higher levels of serum alpha-carotene preconception were associated with shorter time to pregnancy in women with prior pregnancy losses [15]. We observed lower vitamin E intake among women reporting infertility. Vitamin E was first discovered as a dietary component essential for reproduction [16]. Evidence suggests the antioxidant actions of vitamin E may support endometrial health among women receiving fertility treatment [17]. Vitamin C, which was lower among women reporting infertility, plays a role in ovulation and uterine function as well as hormone production [18,19]. A prior study of women with sufficient vitamin B6 found higher odds of conception and less risk of early pregnancy loss in conception cycles, while low vitamin B6 status may reduce conception chances and increase early pregnancy loss risk [20]. Although, a Cochrane review of randomized clinical trials concluded antioxidant supplements were not associated with improved outcomes for women undergoing assisted reproductive procedures techniques [21]. Studies suggest vitamin K deficiency has negative impacts on spermatogenesis [22,23], but the potential role of vitamin K for supporting female fertility is unclear.

Overall, women who experienced infertility had gaps in the intake of multiple nutrients that are important for conception, overall health, and for maternal health and fetal growth and development should they become pregnant [11,24]. Nutrient gaps were reduced with dietary supplementation but persisted, highlighting the need for public health action to improve dietary intake. Currently, dietary guidance focusses on averting nutrient deficiencies rather than on supporting optimal health. The nutritional needs of women during the pre-conception period are thus not currently part of public health guidelines and there are no specific dietary intake requirements unlike those for pregnancy. Our findings suggest antioxidant nutrients; vitamins A, C and E, and selenium in addition to vitamins B6, D and K, magnesium, potassium, lutein and zeaxanthin may warrant focus in future efforts aimed at delineating the nutritional requirements to support fertility.

A strength of this study is the use of multiple cycles of NHANES with sampling weights which permitted estimation of nationally representative dietary intake. Usual intake modelling was a further strength, providing a more accurate estimate of nutrient-fertility relationships, than daily nutrient intake, given that infertility is generally defined as difficulty conceiving over at least one year. We were, however, limited by the assessment of dietary intake by self-report which is prone to measurement error. The assessment of fertility was a further limitation. No context as to the length of time trying to conceive or length of time to pregnancy was available. It was thus not possible to distinguish between primary infertility (inability to conceive a pregnancy ever) and secondary infertility (inability to become pregnant after a successful prior pregnancy) which may differ in etiology and risk factors. The recency of infertility was not captured in the NHANES questionnaire and it is possible that women experienced infertility years before the assessment of dietary intake. This may result in misclassification which could bias the findings towards the null. Due to the cross-sectional study design, we cannot determine temporality of associations. That is, women may have also changed their dietary intake and other related health behaviors as a result of experiencing infertility rather than nutrient gaps contributing to infertility. Prospective studies are needed to model associations between nutrient intake and risk of infertility and determine the temporality of nutrient-fertility relationships.

## Conclusions

Adequate nutrient intake plays a crucial role in reproductive health. Findings from this study highlight significant nutrient inadequacies in women experiencing infertility and provide a starting place for future studies to examine fertility and intake of vitamins A, C and E, B6, D and K, selenium, magnesium, potassium, lutein and zeaxanthin. Our results fill a gap in evidence on nutrient intake among women of childbearing age who experience infertility. Replication of these findings in additional studies and populations may help inform management of infertility in the future.

## Abbreviations:

AI: adequate intake
BMI: body mass index
DRI: dietary reference intake
DHA: docosahexaenoic acid
EAR: estimate average requirement
EPA: eicosapentaenoic acid
NHANES: National Health and Nutrition Examination Survey
